# Pathogenic Roles of Glutamic Acid Decarboxylase 65 Autoantibodies in Cerebellar Ataxias

**DOI:** 10.1155/2017/2913297

**Published:** 2017-03-12

**Authors:** Hiroshi Mitoma, Mario Manto, Christiane S. Hampe

**Affiliations:** ^1^Medical Education Promotion Center, Tokyo Medical University, Tokyo, Japan; ^2^Unité d'Etude du Mouvement (UEM), GRIM, FNRS, ULB Erasme, 1070 Bruxelles, Belgium; ^3^School of Medicine, University of Washington, Seattle, WA 98109, USA

## Abstract

Reports suggesting a pathogenic role of autoantibodies directed against glutamic acid decarboxylase 65 (GAD65Abs) in cerebellar ataxias (CAs) are reviewed, and debatable issues such as internalization of antibodies by neurons and roles of epitopes are discussed. GAD65 is one of two enzymes that catalyze the conversion of glutamate to the inhibitory neurotransmitter gamma-aminobutyric acid (GABA). A pathogenic role of GAD65Ab in CAs is suggested by in vivo and in vitro studies. (1) Intracerebellar administration of cerebrospinal fluid (CSF) immunoglobulins (IgGs) obtained from GAD65Ab-positive CA patients impairs cerebellar modulation of motor control in rats. (2) CSF IgGs act on terminals of GABAergic neurons and decrease the release of GABA in cerebellar slices from rats and mice. (3) Absorption of GAD65Ab by recombinant GAD65 diminishes the above effects, and monoclonal human GAD65Ab (b78) mimic the effects of CSF IgGs in vivo and in vitro. Studies using GAD65-KO mice confirm that the target molecule is GAD65. (4) Notably, the effects of GAD65Ab depend on the epitope specificity of the monoclonal GAD65Ab. Taken together, these results indicate that epitope-specific GAD65Ab-induced impairment of GABA release is involved in the pathogenesis of GAD65Ab-positive CA and support the early detection of GAD65Ab-associated CA to initiate immunotherapy before irreversible neuronal death in the cerebellum.

## 1. Introduction

In addition to the hippocampus, the cerebellum is one of the main targets of autoimmunity in the central nervous system (CNS). Cerebellar damage leads to the development of cerebellar ataxias (CAs), a group of disorders characterized by motor incoordination and impaired cognitive operations [1, 2]. In the last 30 years, the new entity of immune-mediated CAs (IMCAs) has emerged. IMCAs include mainly paraneoplastic cerebellar degeneration, gluten ataxia, and glutamic acid decarboxylase (GAD) 65-antibody associated CA (GAD65Ab-associated CA) [2, 3]. Recent clinical studies suggest a higher than expected incidence of IMCAs and prospective studies by Hadjivassiliou et al. in the UK [4] indicate that the prevalence of IMCAs is 32% in a study of 320 patients with sporadic CAs. IMCAs respond to various immunotherapies, and the response correlates inversely with the latency between onset of CAs and initiation of treatment [3, 5]. Thus, early diagnosis and treatment of IMCAs are important aspects of clinical management of CAs. Therapies of these disabling disorders should take into account the specific pathogenesis affecting cerebellar circuitry [6].

Considering the various types of IMCAs, the pathomechanisms of CAs have been best elucidated for GAD65Ab-associated CA. However, the involvement of Abs in the pathogenesis of CAs has been the subject of intense debate [2]. A pathological role of GAD65Ab was initially rejected based on (1) the intracellular localization of GAD65 and (2) the association of GAD65Ab with various types of neurological diseases, including CAs, stiff-person syndrome (SPS), and epilepsy [2]. However, recent studies challenge this view as they clearly show that monoclonal GAD65Ab interfere with GABAergic neurotransmission in slice preparations and elicit neurophysiological and behavioral effects in animals that mimic CAs in vivo [7]. As recently reviewed by Lancaster and Dalmau, neuronal autoantigens include nuclear and cytoplasmic proteins (group 1), cell surface proteins, specifically synaptic proteins, (group 2) and intracellular synaptic antigens (group 3) [8]. T-cell mediated immune responses are considered to be the pathogenic mechanisms of neurological diseases in group 1, while autoantibodies directed to cell surface proteins in group 2 may cause encephalitis. For disorders associated with intracellular synaptic antigens of group 3, such as GAD65 and amphiphysin, both T-cells and autoantibodies have been implicated in the pathogenesis.

The present review focuses on studies which support pathogenic roles of GAD65Ab in CAs and will discuss open questions, in particular the issue of the penetration of Ab in neurons and the roles of epitopes. We also address the clinical pathophysiological features of GAD65Ab-associated CAs and physiological roles of GABAergic neurons in cerebellar motor coordination.

## 2. Clinical Profiles of GAD65Ab-Associated CA

GAD65Ab-associated CA was first described in a few case studies [9, 10], followed by a systematic survey of 14 patients leading to the establishment of this clinical entity [11]. The condition affects mostly women in their 60s. GAD65Ab-associated CA can be subacute or chronic in terms of clinical presentation. Patients exhibit gait and posture deficits, dysarthria, and nystagmus. An association with type 1 diabetes mellitus (T1DM) is frequently observed [10–14]. Notably, brain MRI of GAD65Ab-associated CA patients reveals only mild or no cerebellar atrophy [11]. CSF analysis shows oligoclonal bands and positivity for GAD65Ab usually without elevation of cell and protein content [11], indicating intrathecal GAD65Ab production, rather than a compromised blood-brain barrier (BBB). Circulating GAD65Ab are often present with titers that exceed those typical for patients with T1DM by 10 to 100-fold [11].

Induction therapies include intravenous methylprednisolone, intravenous immunoglobulin (IVIg), plasma exchange, and rituximab to reduce the severity of symptoms, followed by maintenance therapy with oral prednisolone, azathioprine, mycophenolate mofetil, or repeated IVIg therapy [3, 5, 11, 15]. Most patients with subacute CA show clinical improvement after long-term therapy, whereas patients with chronic CA often show poor response with limited improvement or even progression of deficits during long-term follow-up [16]. The observation that amelioration of CA-related clinical signs and symptoms following immunotherapy is associated with simultaneous decrease in GAD65Ab titers is an additional argument for a pathologic role of these autoantibodies [3, 17]. Finally, GAD65Ab-associated CAs are sometimes reported in paraneoplastic conditions [18].

## 3. Physiology of GAD65 and Cerebellar GABAergic Neurons

### 3.1. Localization and Physiological Roles of GAD65

GAD catalyzes the conversion of glutamate to *γ*-aminobutyric acid (GABA), the main inhibitory neurotransmitter in the CNS. GAD is expressed in CNS inhibitory neurons and pancreatic *β* cells. There are two isoforms of GAD, namely, GAD67 and GAD65 [2, 19]. GAD67 is present mainly in the cytoplasm of inhibitory neurons and regulates the basal level of GABA [19]. The smaller isoform GAD65 is anchored to synaptic vesicles [20, 21]. GAD65 mediates transient GABA synthesis in response to acute demand and facilitates the transport of GABA-containing synaptic vesicles from the Golgi apparatus to the synaptic terminals [22].

### 3.2. Physiological Roles in GABAergic Neurons in Cerebellar Motor Control

**Figure 1:**
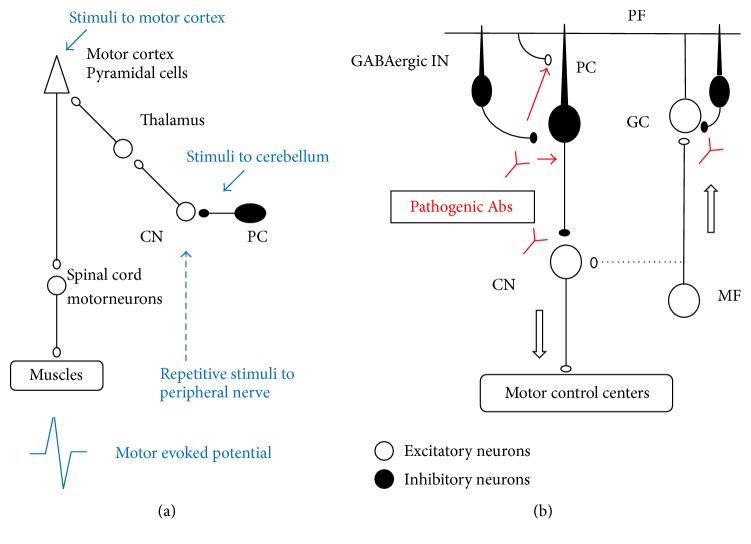
(a) Schematic diagram of electric stimulation experiment in vivo. (b) Schematic diagram of effects of GAD65Ab in the cerebellar circuit. PC: Purkinje cell, GC: granule cells, PF: parallel fiber, MF: mossy fiber, GABAergic IN: GABAergic interneuron, CN: Cerebellar nuclei. Arrows in (b) indicate the flow of signals in the cerebellar cortex. The CN neurons receive weak MF collaterals in the cerebrocerebellum and strong collaterals in other areas. Thus, the MF collateral is indicated by a dotted line. These figures were modified from our previous references [7, 30, 31].

Recent physiological studies have clarified the principles in the cerebrocerebellar loops that coordinate voluntary movements. Importantly, chained GABAergic inhibitory neurons (inhibitory interneurons and Purkinje neurons) work mainly within the cerebrocerebellar loops, since dentate nucleus neurons (DNs) have weak mossy fiber collaterals (see Figure 1(b)) [23, 24].

Studies in monkeys have shown how cerebellar cortex neurons are activated at the initiation of a particular movement [23, 24]. During wrist movement, Purkinje cells (PCs) with somatosensory receptive fields (RFs) in the distal arm are strongly suppressed before movement onset, while DNs with the same RFs show concurrent bursts of activity (disinhibitory control on DNs). In contrast, PCs with RFs in the proximal arm show marked and simultaneous increase in activity, while DNs with the same RFs are strongly depressed (inhibitory controls on DNs) (see diagram in Figure 1(b)) [23, 24]. Through this “disinhibition/inhibition mode,” one can dexterously manipulate an object using the hand with the fixation of proximal muscles.

This dual mode highlights the importance of exact timing in activities of chained GABAergic neurons. Deficits in GABAergic neurons impair neuronal control, producing inappropriate strength/duration in the cerebellar output and resulting in impairment of cerebellar modulation of the motor cortex [24].

By contrast to the cerebrocerebellar loop, the physiological role of GABAergic neurons might be different in the spinocerebellar loop that controls truncal muscle activities. In the spinocerebellar loop, deep cerebellar nuclei receive excitatory input from the spinal cord via collaterals of the mossy fibers [23, 24], and GABAergic outputs from PCs appear to modulate the main pathway constituted of mossy fiber-deep cerebellar nuclei neurons.

## 4. Evidence Suggesting Pathogenesis of GAD65 in Development of Cerebellar Ataxias

While the association between GAD65Ab and CAs is known, the prevalence of GAD65Ab-associated CA is currently unclear. Accumulating evidence indicates that GAD65Ab impair cerebellar GABAergic synapses, leading to clinical manifestations of CAs. In this review, these findings are discussed from in vivo and in vitro levels to molecular level as follows:

1. *Passive Transfer Experiments*. Induction of symptoms of ataxic disorders in vivo by administration of autoantibodies present in GAD65Ab-associated CA patients.
2. *Corresponding Physiological Effects*. Impairment of GABAergic neurotransmission in vitro in cerebellar circuit preparations by the above antibody preparations.
3. *Identification of Pathogenic Abs*. Identification of GAD65Ab as the pathogenic fraction in CSF IgGs obtained from GAD65Ab-associated CA patients.
4. *Epitope Specificity*. Identification of distinct GAD65Ab epitope specificities in GAD65Ab-associated CA patients that induce responses similar to those observed for CSF IgGs.

### 4.1. Passive Transfer Experiments

Passive transfer experiments are critical to confirm the pathogenicity of GAD65Ab in CAs and demonstrated GAD65Ab-mediated impairment of cerebellar modulation of motor control and GAD65Ab-mediated molecular changes.

*Impairment of Cerebellar Modulation of Motor Control. *Cerebellar modulation of motor cortex excitability can be examined by measuring the response (motor evoked potential, MEP) evoked in the gastrocnemius muscle to stimulation of the contralateral motor cortex (representing an index of motor cortex excitability) [25, 26]. Experimentally, potentiation of the corticomotor response is induced by preceding trains of repetitive stimuli applied to a peripheral nerve [25, 26]. The cerebellum is involved in this potentiation since removal of the cerebellar hemisphere impairs this short-term potentiation [27]. In addition to MEPs, the effects of the cerebellar hemisphere on contralateral motor cortex can be examined using a protocol of cerebellocortical inhibition (CCI). A preceding stimulus over the cerebellum inhibits the corticomotor response. That is, MEPs decrease in magnitude when stimulation of a cerebellar hemisphere is applied 2.1 msec before stimulation of the contralateral motor cortex [26]. Mechanistically, stimulation of the inhibitory cerebellar PCs inhibits motor cortex activity through the deep cerebellar nuclei-thalamus-motor cortex pathway (see diagram in Figure 1(a)) [23–27]. The inhibition of cerebellar cortex upon cerebellar nuclei is a key mechanism of cerebellar circuitry [23–27].

Intracerebellar administration of IgGs obtained from CSF of patients with GAD65Ab-associated CA diminishes both cerebellar modulations (MEPs and CCI), suggesting that the CSF IgGs from these patients impair cerebellar modulation of motor control and contribute to the lack of coordination.

*Possible Molecular Changes Leading to Cerebellar Atrophy. *The pathogenic mechanisms leading to cerebellar atrophy have been examined in vivo [25]. The N-methyl-D-aspartate- (NMDA-) mediated calcium entry at postsynaptic densities is coupled with nitric oxide (NO) synthesis [28], which in turn inhibits glutamate release, thereby preventing excitotoxic neuronal death [29]. Microdialysis in rat cerebellum has been used to measure NMDA-mediated production of NO and NMDA-mediated regulation of glutamate. Intracerebellar administration of CSF IgGs specifically reduces the NMDA-mediated production of NO and impairs the synaptic regulation of glutamate after NMDA administration, thereby potentially facilitating pathological processes that lead to cerebellar atrophy by excitotoxicity [25].

### 4.2. Corresponding Physiological Effects in Cerebellar Circuits

The pathogenic effects of CSF IgGs in cerebellar circuits have been examined using whole-cell recordings from isolated rat and mice cerebellar slices [7, 30–34].

**Figure 2:**
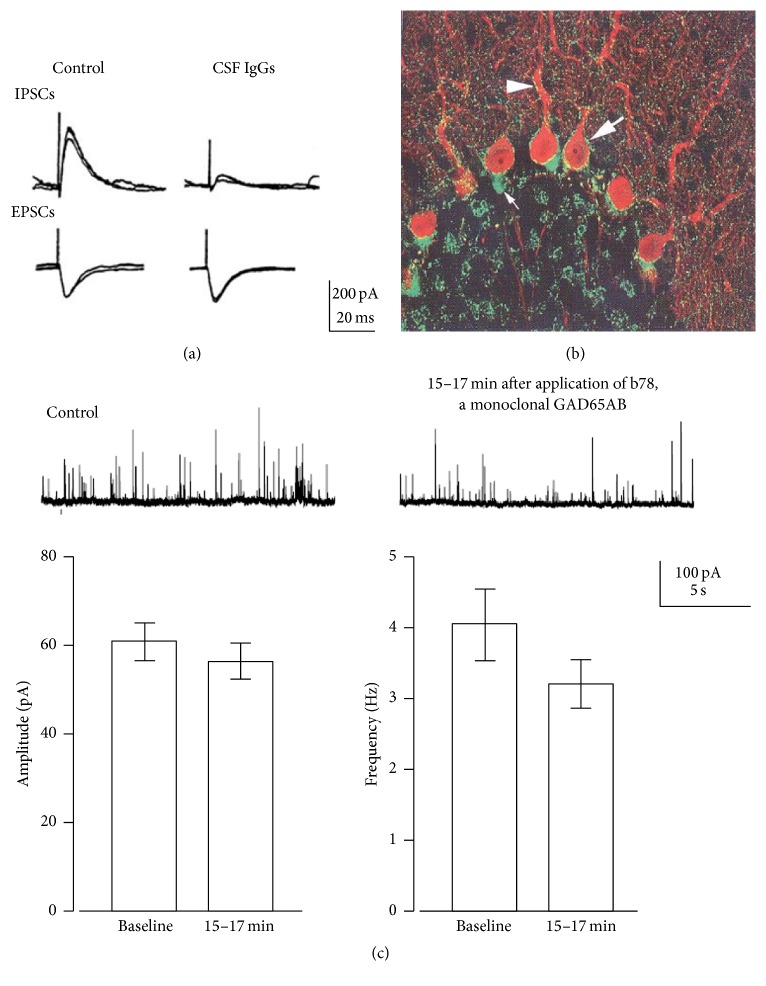
Pathogenic actions of CSF IgGs from GAD65Ab-positive CA and human monoclonal GAD65Ab b78 in cerebellar slices prepared from rats and mice. (a) Electrical stimulation in the cerebellar cortex induces GABA-mediated inhibitory postsynaptic currents (IPSCs) and glutamate-mediated excitatory postsynaptic currents (EPSCs) in a PC. The CSF IgGs depressed IPSCs without affecting EPSCs. Two sweeps are superimposed. (b) Immunoreactivities of CSF IgGs were observed in the presynaptic terminals of GABAergic interneurons and not the postsynaptic PC. Large and small white arrows indicate punctate immunoreactivity in the rim of the somata and high density of immunoreactivity terminals in the initial segment of PC, respectively. Arrowhead indicates immunoreactivity along with dendritic shaft. (c) b78-induced impairment in GABA release: decreased amplitudes of miniature IPSCs, reduced GABA content in vesicles, and reduction of frequency of miniature IPSCs, and the release probability of GABAergic vesicles. Two sweeps (grey and black) are superimposed. Data are mean ± SEM of 12 experiments. These figures were modified from our previous references [7, 30, 31].

*Effects of IgGs on Cerebellar GABAergic Synaptic Transmission*. Application of CSF IgGs fractions from patients with GAD65Ab-associated CA selectively suppresses inhibitory transmission from basket cells, that is, GABAergic interneurons, to PCs, without affecting excitatory transmission (Figure 2(a) and diagram in Figure 1(b)) [7, 30–34]. The CSF IgGs suppress inhibitory postsynaptic currents (IPSCs) for more than 1 hour after termination of 8–10 minutes of CSF application (Figure 2(a)).

The immunohistological finding that CSF IgGs react predominantly with the presynaptic terminals of GABAergic interneurons and not with the postsynaptic PCs (Figure 2(b)), together with the observation that inhibition of the paired-pulse ratio during the inhibitory phase by CSF IgG mimics that of presynaptic inhibitors (low Ca^2+^) but not postsynaptic antagonists (bicuculline) [31, 32], has led to the conclusion that the CSF IgG-mediated synaptic depression is mediated by presynaptic mechanisms.

The decrease in GABA release consequently elicits hyperexcitability of PCs in cerebellar circuits [33]. GABA released from basket cells not only produces IPSCs on PCs through GABA_A_ receptors but also accesses neighboring excitatory glutamate synapses by diffusion, thereby presynaptically inhibiting the release of glutamate by GABA_B_ receptors [33]. Thus, CSF IgGs elicit dual impairments: depression of GABA_A_ receptor-mediated inhibitory synaptic transmission and attenuation of GABA_B_ receptor-mediated inhibition of excitatory transmission (see diagram in Figure 1(b)) [33].

These series of in vitro studies demonstrated that CSF IgGs obtained from patients with GAD65Ab-associated CA act on nerve terminals of GABAergic interneurons to depress the release of GABA, resulting in hyperexcitability of PCs.

### 4.3. Identification of GAD65Ab as the Culprit Pathogenic Abs

*Pathogenic Effects of GAD65Ab*. Because the CSF of CA patients may contain Abs of specificities other than GAD65, it is necessary to exclude the possibility that the above observed pathological changes are caused by other unidentified Abs. The pathogenic role of GAD65Ab in the impairment of GABA release at cerebellar synapses and ataxic disorders in vivo has been confirmed in two sets of experiments. CSF IgGs-induced synaptic depression is completely abolished by absorption of GAD65Ab by recombinant GAD65 [34]. Furthermore, human monoclonal GAD65Ab b78 elicits pathogenic effects similar to those induced by CSF IgGs [7, 26]. Application of b78 reduces the amplitude of IPSCs with a long-term time course in the cerebellar circuits [7]. Intracerebellar application of b78 abolishes cerebellar modulation of motor cortex in in vivo preparations [7, 26] and also induces ataxic gait and cognitive dysfunction in rodents [7, 35].

*GAD65 Molecule as the Target Molecule*. To confirm that GAD65 is the sole target of GAD65Ab, we have examined the effect of human monoclonal GAD65Ab b78 administration in GAD65 knockout (GAD65-KO) mice [7]. The effects of b78 on inhibitory synaptic transmission were compared between cerebellar slices obtained from wild-type mice, where IPSCs are mediated by GAD65 and GAD65-KO slices, where IPSCs are mediated compensatorily by GAD67. Synaptic depression was observed only in wild-type slices, but not in GAD65-KO slices [7], suggesting that depression of GABA release in wild-type mice is mediated by impairment of GAD65 function.

Taken together, these in vivo and in vitro physiological studies show that binding of GAD65 by GAD65Ab elicits loss of GAD65 functions pertaining GABA release, leading to the development of CAs.

### 4.4. Epitope Specificity

*Epitope Specificity of GAD65Ab*. GAD65Ab are observed not only in patients with CAs but also in stiff-person syndrome (SPS) and type 1 diabetes (T1DM) patients [36, 37]. Earlier studies have demonstrated a difference in both GAD65Ab titer and epitope specificity, as GAD65Ab titers in SPS exceed those in T1DM by up to 500-fold and also recognize linear epitopes, while GAD65Ab in T1DM are strictly dependent on the conformation of the antigen [38–40]. Moreover, only CA and SPS patients show distinct neurological symptoms. To investigate whether these differences in clinical phenotypes were due to specific GAD65Ab localization (SPS and CA patients present GAD65Ab both in the periphery and the CNS [41], while GAD65Ab in T1DM patients are only found in the periphery [42]) or to distinct GAD65Ab characteristics, studies with human monoclonal GAD65Ab with diverse epitope specificities were conducted [7, 26, 35].

**Figure 3:**
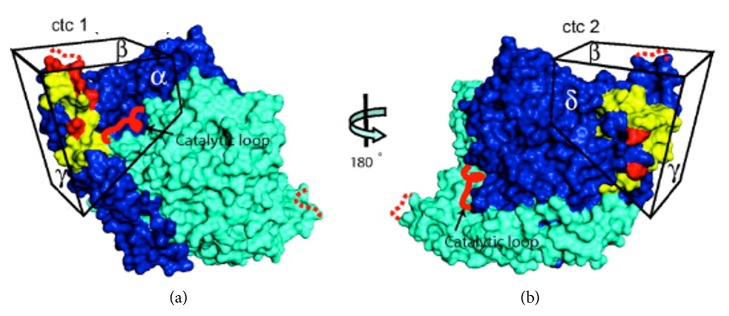
Surface structure of GAD65 showing epitope clusters ctc1 and ctc2. Monomers A and B of the dimeric GAD65 are shown in dark blue and cyan, respectively. Epitope clusters ctc1 and ctc2 on opposing faces of the C-terminal domains are shown. In (b), the molecule is rotated 180° along the vertical axis, and different faces of the C-terminal domain are shown as *α*, *β*, *γ*, and *δ* faces. Broad epitope locations are shown in yellow and single contact sites for mAb binding in red. The disordered catalytic loop and the COOH-terminal flexible loop are represented by red and red dotted lines, respectively. The epitope bound by GAD65Ab b78 resides primarily on the *α* and *β* faces of ctc1, while GAD65Ab b96.11 recognizes an epitope located on the *δ* and *γ* faces of ctc2. This figure was adapted from Fenalti et al. (2008) with permission [44].

Human monoclonal GAD65Ab b96.11 binds to a common epitope that is shared by GAD65Ab in patients with T1DM [26, 43], while human monoclonal GAD65Ab b78 recognizes an epitope that is often recognized by GAD65Ab in patients with SPS and CA [7, 26]. Notably, the b78-defined epitope is rarely recognized by GAD65Ab in patients with T1DM [19]. While both monoclonal GAD65Ab recognize epitopes spanning the middle and C-terminal region of GAD65, the respective conformational epitopes are distinctly different [44] (Figure 3).

Monoclonal GAD65Ab b78 and b96.11 show distinct effects both in vitro and in vivo, as summarized in Table 1 [7, 26]. Importantly, in accordance with the above finding of similar GAD65Ab epitopes recognized by GAD65Ab in CA patients and b78, the effects of CA GAD65Ab on cerebellar functions are reproduced by those of b78 but not by b96.11, both in in vitro and in vivo preparations (Table 1) [7, 26]. These results suggest that neurological impairments caused by GAD65Ab vary according to epitope specificity and could explain the diversity of neurological symptoms in patients with GAD65Ab.

*Primary Impairment Caused by Monoclonal GAD65Ab b78*. The similar effects induced by CA-GAD65Ab and b78 may suggest that the primary impairment is caused by binding of a shared GAD65 epitope. Notably, b78, and not b96.11, interfere with the association of GAD65 and the cytosolic face of GABA-containing vesicles [7]. The dissociation of GAD65 from GABAergic vesicles may impact both packaging of GABA into vesicles and shuttling of GABAergic vesicles to the synaptic cleft [7]. In agreement with this assumption, application of b78 causes dual effects in slice preparations: (1) decreased amplitude of miniature IPSCs, suggesting a reduced GABA content in the vesicles, and (2) reduced frequency of miniature IPSCs, suggesting lower release probability of GABAergic vesicles (Figure 2(c)) [7]. Thus, GAD65Ab-induced dual impairment in the packaging and the shuttling might be the primary change that results in the development of CAs. Accordingly, CSF IgGs from CA patients have no effect on the enzyme activity of GAD65 but interfere with exocytosis, as measured by glycerol turnover [7].

## 5. Open Questions

### 5.1. Physiological Interaction of GAD65Ab with Cytosolic GAD65

GAD65 is mainly located on the cytosolic face of vesicles together with the vesicular GABA transporter VGAT [22]. Thus it has been claimed that the intracellular localization of GAD65 makes it unlikely for GAD65Ab to play a pathogenic role in CAs. While some reports failed to observe internalization of GAD65Ab by live neurons [45], PCs in rat organotypic cultures clearly incorporate both host and nonhost immunoglobulins [46], and kappa and lambda light chains were detected in PCs of a patient with multiple myeloma [47]. Subsequently, internalization of IgGs has been confirmed in the rat cerebellum [48], in autopsied human cerebellar tissue [49], after interventricular injection of human IgGs in guinea pigs [50], and after peripheral administration of human IgGs in rats [51] (Table 2). We have confirmed the internalization of human monoclonal GAD65Ab b78 by cultured AF5 cells [35]; moreover, b78 has been detected in CA1 interneurons and PCs shortly after its injection in the medial septum/diagonal band and ipsilateral interpositus nucleus, respectively [7, 52]. Differences in neuronal cell types, antibody concentration, and detection methods may account for these contradicting results.

In conclusion, the above results strongly suggest that neuronal uptake of immunoglobulin is more frequent than previously recognized. This understanding is also supported by the recent observation that SPS-associated autoantibodies (directed to amphiphysin) are taken up by hippocampal neurons [53]. However, unequivocal evidence about the internalization mechanism route is currently missing. Alternatively, GAD65Ab may also interact with GAD65 during exocytosis, when the antigen is temporarily exposed and thus accessible to GAD65Ab [20, 21].

### 5.2. Heterogeneity of Neurological Manifestations

The association of GAD65Ab with different clinical neurological phenotypes as present in CAs, SPS, and epilepsy [11, 45] has challenged the notion of a pathogenic role of GAD65Ab. Moreover, recent studies cast doubt on the hypothesis that recognition of disease-specific epitopes may induce different clinical phenotypes [54]. In these studies, GAD65Ab epitope specificity in patients with SPS, CA, or epilepsy was investigated using GAD65 fragments.

However, epitope regions of disease-specific monoclonal GAD65Ab often include amino acids present in both the middle region and the C-terminus [44]. Indeed, an earlier study demonstrated a significant reduction of binding of isolated GAD65 fragments by disease-specific monoclonal GAD65Ab [55], thus supporting the argument that epitope mapping using GAD65 fragments may not reflect important binding specificities. Finally, disease-specific effects have been demonstrated also for CSF from SPS patients [26, 41] and CSF from epilepsy patients [56], and a recent study demonstrated significant differences in terms of epitope recognition and induced effects between GAD65Ab in CA and SPS patients [26].

As outlined earlier, GAD65 performs two distinct functions in GABAergic neurotransmission, namely, the facilitation of GABA release and GABA synthesis. Previous studies demonstrated that GAD65Ab present in SPS patients decrease GABA synthesis [26, 38, 41], while GAD65Ab present in CA do not affect GAD65 enzyme activity but may interfere with GABA release, either by inhibiting the packaging of GABA into vesicles and/or by impeding with the subsequent shuttling of vesicles to the synaptic cleft [7, 26] (Table 1). This disease-specific inhibition pattern may have distinct consequences on the associated clinical phenotypes. Roles of GAD65 might be different between the cerebrocerebellar loop, where deficits are closely related to GAD65Ab-associated CA, and the spinocerebellar loop, where dysfunctions are closely related to muscle tonus deficits seen in SPS (see discussions in the previous section of Physiology of GAD65 and Cerebellar GABAergic Neurons). In the cerebrocerebellar loop, the chained GABAergic neurons (inhibitory neurons and PCs) determine the phasic command about timing for coordination [23, 24], with a specific emphasis on the exact timing of GABA release. By contrast, in the spinocerebellar loop [23, 24], GABAergic outputs from PCs modulate excitatory signals, with a specific emphasis on a tonic supply of GABA. These physiological data suggest that GAD65Ab could elicit CAs or SPS depending on the epitope specificity. In accordance with this assumption, administration of CA CSF IgGs into the cerebellar nuclei caused deficits in cerebellar control on motor cortex, whereas that of SPS CSF IgGs elicited hyperexcitability of the spinal cord without affecting control on the motor cortex (Table 1) [26]. These data do not exclude the involvement of autoantibodies directed to other autoantigens, such as amphiphysin, in the pathogenesis of SPS [53].

Further studies are needed to clarify whether manifestations of different neurological symptoms can be attributed to the epitope specificity in GAD65Ab.

### 5.3. Diverse Mechanisms Underlying Cell Death

**Figure 4:**
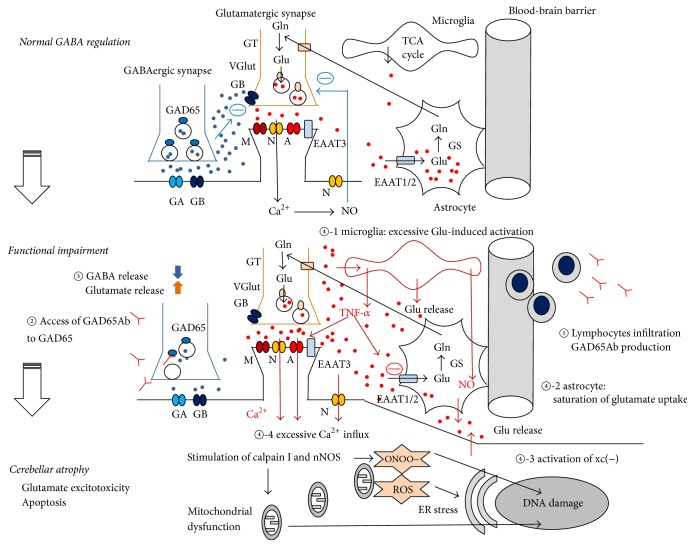
Overview of the possible synaptic consequences of a decreased GABA release in the cerebellum. In normal conditions, GABA released from GABAergic neurons bind to GABA_A_ receptors located on PC and induce an IPSC. GABA also diffuses out the synapse and binds to GABA_B_ receptors, reducing release of the excitatory neurotransmitter glutamate. Glutamate ultimately activates NMDA receptors on excitatory neurons and is regulated in part through NO. Glutamate is taken up by astrocytes via the EAAT pathway. When GAD65Ab is produced intrathecally (*①*), GAD65Ab may bind GAD65 and interfere with GABA release (②). Reduced GABA levels increase glutamate levels as a consequence of lower inhibition of GABA_B_ receptors (③). Continuously high glutamate levels can develop divergent effects as detailed in the following (④). Glutamate activates microglia, which in turn releases more glutamate (④-1). Saturation or impairment of EAAT will reduce glutamate reuptake by astrocytes (④-2). Activation of xc(−) increases the extracellular glutamate release (④-3). Finally, the NMDA/NO feedback regulation fails. All these effects result in an increase in glutamate concentrations. The excessive Ca^2+^ influx stimulates calpain I and nNOS, leading to mitochondria dysfunction, ER stress, and DNA damage (④-4). In this scheme, Purkinje cells and granule cells are assumed. However, NMDA receptors are expressed in granule cells, whereas metabotropic glutamate receptors are expressed in Purkinje cells. Mossy fibers strongly excite granule cells via NMDA and AMPA receptors, contributing to cerebellar neuronal hyperactivity. GA: GABA_A_ receptors, GB: GABA_B_ receptors, GAD: glutamate decarboxylase, A: AMPA receptors, N: NMDA receptors, M: metabotropic glutamate receptors, EAAT: excitatory amino acid transporters, blue dots: GABA, red dots: glutamate, Glu: glutamate, Gln: glutamine, GS: glutamine synthetase, GT: glutaminase, VGlut: vesicular glutamate transporter proteins, and (—): inhibitory effects.

A series of in vitro and in vivo experiments show that GAD65Ab elicit decreased GABA release, thereby reducing GABA_B_ receptor-mediated inhibition of glutamate release. Although this imbalance is assumed to elicit excitotoxicity in cerebellar neurons, detailed mechanisms underlying prominent loss of cerebellar neurons [57, 58] have not been elucidated. In the following section, we will review diverse glutamate-associated mechanisms leading to cell death (see Figure 4).

*(1) NMDA Receptors*. NMDA receptors are a major glutamate receptor class that are found in the cerebellum on granule cells, molecular layer interneurons, and cerebellar nuclei cells [59]. It has been considered, until recently, that excessive glutamate release from presynaptic sites activates an excessive number of postsynaptic NMDA receptors, thus triggering excitotoxic neuronal death by allowing excessive Ca^2+^ influx through receptor-operated cation channels [60]. However, extrasynaptic NMDA receptors contribute actively to the excitotoxic neuronal death [61]. In case of excessive activation of NMDA receptors, a Ca^2+^ influx results in stimulation of calpain I and nNOS [60]. This causes damage to DNA and formation of ONOO− following an excess of NO (nitrosative stress), as well as other free radicals altering mitochondria functions. Overactivation of NMDA receptors by high glutamate levels can cause excitotoxicity, as demonstrated experimentally on granule cells [59].

The contribution of NO is complex. Under normal conditions, activation of NMDA receptors is coupled with NO synthesis, which in turn inhibits further glutamate release as a compensatory mechanism as explained earlier (Figure 4) [25]. However, in vivo studies show that CSF IgGs obtained by GAD65Ab-positive patients impair this negative-feedback regulation [25]. The continuous lack of GABA_B_ receptor activation eventually leads to a decrease in NMDA receptors [62], with a potential impact upon NO production.

*(2) Microglia*. Recent studies have shown that excessive glutamate levels stimulate microglia [63], which in turn facilitates release of glutamate [64] and proinflammatory cytokines that alter synaptic transmissions (Figure 4) [65]. Thus, the cross talk between glutamate and microglia might also be involved in the positive feedback loop that accelerates hyperexcitability in cerebellar neurons. Indeed, microglia release large amounts of TNF-*α*, which is an important component of the neuroinflammatory response [66]. TNF-*α* potentiates glutamate-mediated cytotoxicity by two complementary mechanisms: an inhibition of glutamate transport on astrocytes (see below) and triggering of the expression of Ca^2+^ permeable-AMPA receptors and NMDA receptors [66]. By contrast, TNF-*α* reduces expression of GABA_A_ receptors on neurons. Thus, the net result is a shift towards excitation. Microglia are also involved in the release of NO. Activation of microglia results in iNOS expression, leading to overproduction of NO (dual role of NO: detrimental or protective to neurons under oxidative toxicity), Ca^2+^ release from the endoplasmic reticulum, and release of vesicular glutamate from glial cells, thus contributing to excitotoxicity [67].

*(3) Astrocytes*. Excitatory amino acid transporters (EAATs), expressed on astrocytes, mediate reuptake of glutamate from the synapse [68]. Excessive levels of glutamate observed after the administration of monoclonal GAD65Ab [25] suggest saturation or impairment in the EAATs-mediated clearance system (Figure 4).

Interestingly, these mechanisms could accelerate a process of excitotoxicity that is triggered by a decrease of GABA release. A similar example of positive feedback is reported in the anti-NMDA receptor Ab-associated encephalitis, a hippocampus autoimmune disease where anti-NMDA Ab block the NMDA/NO-mediated inhibition of glutamate release [69]. This spiral of positive feedback might be one reason why the cerebellum and hippocampus are vulnerable organs for autoimmune attacks.

*(4) The System xc(−)*. xc(−) is a cystine/glutamate antiporter exchanging extracellular cystine for intracellular glutamate [70]. By a direct effect upon intracellular contents of cysteine/GSH, the system xc(−) is a regulator of the antioxidant pathway. Importantly, in several brain regions, system xc(−) is a key-source of extracellular glutamate. The transcription of xCT, a subunit of system xc(−), is increased in the presence of reactive oxygen species (ROS including the superoxide anion O^2−^, the hydrogen peroxide H_2_O_2_, and hydroxyl radicals OH^·^, causing damage to lipids, proteins, and DNA) and proinflammatory cytokines, thus contributing to extracellular glutamate release in neurological conditions associated with neuroinflammation.

Taken together, functional impairments could result in cerebellar atrophy through divergent mechanisms. Therefore it is critical that induction therapy should be applied during the earlier stages of functional impairment. At an early phase, the cerebellar feed-forward control is still preserved, probably because damage caused by GAD65Ab is limited to functional synaptic impairment [2, 6]. Conversely, in the stage of cerebellar atrophy, reversal of cerebellar symptoms is difficult, although immunotherapy may prevent further advancement of the autoimmune processes [6, 15].

## 6. Conclusions

The hypothesis of a pathological role of GAD65Ab was initially rejected based on the intracellular distribution of the target antigen and the association of GAD65Ab with a spectrum of neurological symptoms. However, physiological experiments in vivo and slice preparations provide substantial evidence that GAD65Ab with distinct epitope specificities interfere with GABA release in cerebellar circuits, resulting in ataxic symptoms. While mechanistic details, including mechanism of internalization of Ab and the symptomatic diversity, remain to be uncovered, these experiments strongly support a pathologic role of GAD65Ab in neurological disorders.

It should be underlined that findings reviewed here do not exclude the involvement of T-cell-mediated mechanisms in GAD65Ab-associated CA. However, they provide substantial evidence that epitope-specific GAD65Ab impair GAD65 function, resulting in diminished GABA release, with the consequent development of clinical manifestations of CAs. The relation between Abs-induced impairment and T-cell-mediated damage needs to be examined in more detail.

*“One to Multiple”: Antibody-Mediated Decreases in Neurotransmitter Levels and Their Consequences.* Pathogenic autoantibodies may affect single or several mechanisms. Autoantibodies targeting voltage gated calcium channels (VGCC) in neuromuscular junctions block calcium influx, thereby decreasing the release of acetylcholine and eventually leading to muscle weakness (Lambert-Eaton syndrome). No other effect of autoantibodies to VGCC has been determined so far. GAD65Ab-mediated decrease in GABA not only reduces stimulation of GABA_A_ receptors but also diminishes the effect of GABA on GABA_B_ receptors located on the terminals of neighboring parallel fibers [33]. Here, GAD65Ab-induced depression in GABA release results in increased glutamate release [33]. Sustained increase in glutamate release triggers subsequent responses, including activation of NMDA receptors, with later impairment in NMDA receptor-mediated responses, activation of microglia, and saturation of EAAT-mediated glutamate uptake by astrocytes. These secondary changes may accelerate the process of excitotoxicity and result in the progression of dysfunctional signal transmission to the stage of cell degeneration. In agreement with this assumption, cerebellar atrophy is evident with the progression of the disease [2]. The autopsy report in a patient with advanced stage CA indicated complete loss of PCs [57, 58]. Thus, GAD65Ab-induced decrease in GABA release characteristically elicits chained and cascaded effects in the cerebellum.

In conclusion, recent studies indicate that GAD65Ab play a pathogenic role in the development of CAs that is dependent on their epitope specificity and that GAD65Ab elicit a decrease in GABA release at cerebellar synapses and functional impairment in cerebellar motor controls. This would be subsequently followed by a cascade of events leading to cerebellar atrophy. These studies also support a clinical recommendation to start induction immunotherapies during the functional disorder stage, thereby necessitating a very early diagnosis.

## Figures and Tables

**Table 1:** Summary of effects of polyclonal and monoclonal GAD65Ab in vitro and in vivo.

		SPS GADAb	CA GADAb	b78	b96.11
In vitro	GABA synapse response in slices	Not done	Inhibition	Inhibition	Transient inhibition
	Association of GAD65 with GABAergic vesicles	Not done	Not done	Impairment	No effect
	Enzyme activity	Inhibition	No effect	Inhibition	No effect

In vivo	Glycerol turnover^*∗*^	No effect	Reduction	Reduction	No effect
	Corticomotor response^¶^	No effect	Impairment	Strong impairment	Impairment
	Cerebellar inhibition of MEPs^¶^	No effect	Decrease	Strong impairment	Decrease
	Premotor facilitations on MEPs^¶^	Not done	Not done	Impairment	No effect
	Conditioned eyelid responses^§^	Not done	Not done	Impairment	No effect
	Gait	Not done	Not done	Ataxic	No effect

^*∗*^Glycerol turnover reflects membrane turnover as a result of exocytosis of GABA-containing vesicles [26].

^¶^Corticomotor response: the preceding trains of repetitive stimuli to peripheral nerve potentiate the amplitude of motor evoked potential (MEP) in normal controls.

^¶^Cerebellar inhibition on MEPs: when the cerebellum is stimulated prior to motor cortex stimulation, MEP decreases in amplitude in normal controls.

^¶^Premotor facilitation on MEPs: when trains of stimuli are delivered to the prefrontal cortex, the paired pulses ratio (conditioned MEP/unconditioned MEP) increases in normal controls.

These protocols examine cerebellar modulations on motor cortex excitability [7, 25, 26].

^§^Classical conditioning of eyelid responses was performed. A short or long tone was used as the conditioning stimulus, and electrical shock was used as non-conditioning stimulus. The learning process of eyelid response is mediated by the cerebellum. Conditioned eyelid responses evoked in b78 mice were smaller than in control mice [7].

These results are summarized from [7, 26].

**Table 2:** Summary of experiments showing internalization of antibodies into cerebellar neurons.

Preparation	Detected internalized antibodies	Portion of internalization	Reference
In vitro incubation of rat cerebellum with human or rat IgGs and IgMs	IgG, IgM	Purkinje cells	[46]
Autopsy Brain tissue of patient with multiple myeloma	Myeloma light chain	Purkinje cells	[47]
Autopsy Brain tissue of rats	IgG	Purkinje cells, specific neuronal nuclei	[48]
Autopsy Cerebellar tissues of patients	IgG, IgA, IgM	Purkinje cells	[49]
Intraventricular injection of human IgGs into guinea pigs	IgG	Purkinje cells	[50]
Peripherally administered human IgG in rats.	IgG	Purkinje cells	[51]
